# Cervical Intramedullary Spinal Cord Abscess Secondary to Discitis and Osteomyelitis in an Immunocompromised Patient

**DOI:** 10.7759/cureus.56477

**Published:** 2024-03-19

**Authors:** Ashlie Maldonado-Pérez, Samuel Estronza, Hiram J Maldonado, Emil A Pastrana, Orlando De Jesus

**Affiliations:** 1 Neurosurgery, University of Puerto Rico, Medical Sciences Campus, San Juan, PRI; 2 Neurosurgery/Critical Care Medicine, University of Puerto Rico, Medical Sciences Campus, San Juan, PRI

**Keywords:** immunocompromised, abscess, spinal cord, intramedullary, osteomyelitis, discitis

## Abstract

Intramedullary spinal cord abscess is a rare neurological condition, not commonly suspected and often misdiagnosed. Even after a prompt diagnosis and treatment, most patients persist with permanent neurological deficits. In adults, factors such as immunocompromised, intravenous drug use, endocarditis, and sepsis could be associated with its development. In this study, we present the case of a 63-year-old male patient who developed a chronic cervical intramedullary spinal cord abscess after being treated for multiple abscesses in the paravertebral and psoas muscles. A diagnosis of cervical intramedullary spinal cord abscess secondary to osteomyelitis and discitis was made. He underwent a two-stage cervical surgery, with drainage of the abscess, spinal stabilization, and intravenous antibiotics. Although rare, vertebral osteomyelitis and discitis may be related to its development. Early diagnosis, prompt abscess drainage, and appropriate antibiotic therapy are of utmost importance to improve prognosis and minimize the long-term sequelae and complications of permanent neurological deficits.

## Introduction

Intramedullary spinal cord abscess (ISCA) is an uncommon diagnosis associated with high mortality if untreated [1]. Prompt recognition and intervention are crucial to offer our patients the best expectations. ISCA frequently presents with motor deficits in about 90% of the cases [1,2]. Other symptoms include sensory alterations, infection signs, and bowel or urinary dysfunction [2]. Magnetic resonance imaging (MRI) is the gold standard for diagnosis. The MRI may show hyperintensity on T2, with a ring-enhancing lesion after contrast enhancement [3]. ISCA may also appear as a cystic cavitation with septation within the spinal cord [3]. As a rare neurological condition, it is not commonly suspected and, thus, is often misdiagnosed. In misdiagnosed cases, where a prompt diagnosis is of paramount importance, most patients had permanent neurological deficits such as paraplegia or profound loss of sensation. A recent literature review found that approximately 20% of the cases presented with chronic ISCA, 29% were acute, and 33% were subacute [2]. We present the case of an immunocompromised male patient who developed a chronic cervical ISCA after being treated for multiple abscesses in the lumbar paravertebral and psoas muscles.

## Case presentation

**Figure 1:**
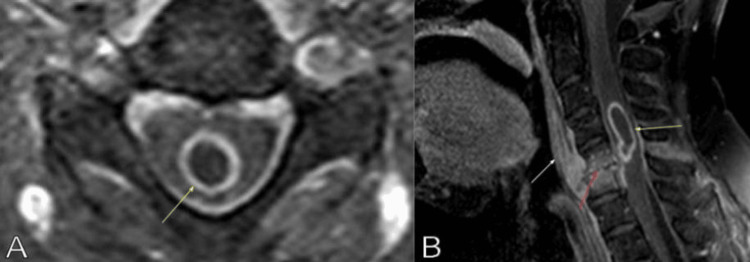
Cervical magnetic resonance imaging T1-weighted with gadolinium. (A) Axial and (B) sagittal images showing the intramedullary abscess (yellow arrow) associated with the C5-C6 discitis and osteomyelitis (red arrow) and the infectious process extending into the prevertebral/retropharyngeal space (white arrow).

A 63-year-old male patient with a past medical history of colon cancer 20 years before was evaluated by the neurosurgery service due to left hemibody weakness. He was diagnosed 30 years before with human immunodeficiency virus after living with multiple same-sex partners. During the previous six months, his primary care physician treated the patient for chronic back pain. However, in the last month, he developed severe acute pain in the lower back region, and a lumbar computed tomographic scan showed multiple abscesses in the left psoas, paravertebral, and obturator muscles for which he was admitted and treated with intravenous antibiotics for three weeks. However, when he was going to be discharged home on oral antibiotics, he noticed difficulty in walking and left-side weakness, for which a brain and cervical MRI were done. The cervical MRI with and without gadolinium showed loss of the C5-C6 intervertebral disc height with osseous destruction of the corresponding vertebral endplates and vertebral body signal abnormalities, consistent with diskitis and osteomyelitis (Figures 1A, 1B). The inflammatory process extended posteriorly to the anterior epidural space and formed an ISCA. The intramedullary abscess measured 2.4x0.9x0.9 cm. Anteriorly, the infectious process also involved the prevertebral and retropharyngeal spaces, with a long segment phlegmonous process, which measured 0.3x1.0x4.2 cm at the levels of C3-C5.

The patient was transferred to our hospital for further evaluation and management by a spine neurosurgeon. He denied recent spinal or nerve block, fever, or chills. The patient was afebrile with stable vital signs. Upon physical examination, there was tenderness in the paraspinal muscles. The right upper and lower extremities showed muscle strength grading 5/5. The left upper extremity was 3/5, with the left lower extremity 4/5. All sensory modalities were intact. The deep tendon reflexes were normal in all the articulations. The complete blood count showed five thousand white blood cells/µL. The erythrocyte sedimentation rate was 53 mm/h, and the C-reactive protein was 16.9 mg/dL. The blood cultures were negative throughout the hospitalization. The CD4 count was 375 cells/mm^3^ of blood.

**Figure 2:**
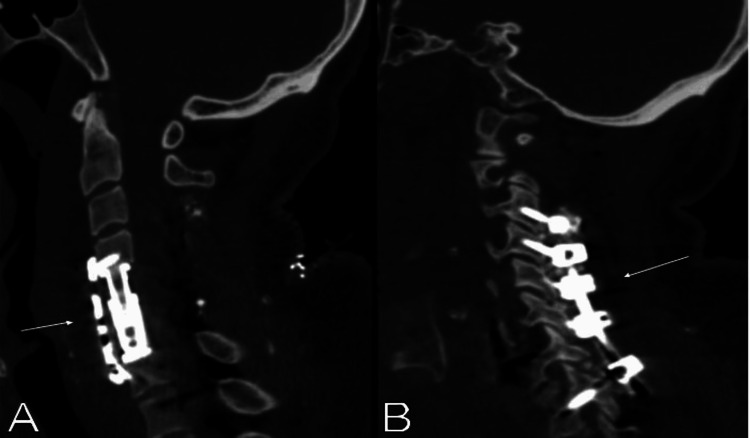
Cervical spine computed tomographic scan sagittal images showing the spinal reconstruction. (A) Anterior C5-C6 corpectomy with an expandable titanium vertebral body replacement cage from C4 to C7 and an anterior titanium plate (arrow) and (B) posterior C3-T1 instrumentation (arrow).

Surgical planning was recommended, consisting of a two-step surgery to maximize success and complete resection of the inflammatory phlegmon and ISCA (Figures 2A, 2B). An anterior C5-C6 corpectomy was performed first. After removal of the posterior longitudinal ligament, purulent material was found in the epidural space, along with inflammatory phlegmon, severely compressing the spinal cord. The phlegmon was carefully resected with complete decompression of the spinal cord. An expandable titanium vertebral body replacement cage was placed from C4 to C7. Then, a posterior approach was performed to drain the ISCA. After a C3-C6 laminectomy, the posterior cervical dura was opened. The spinal cord was opened at the paramedian plane, allowing drainage of the abscess cavity. A posterior C3-T1 instrumentation was done.

Infectious diseases service recommended intravenous cefepime 2 g every 8 h and vancomycin 1.5 g every 12 h. Four days after starting antibiotic therapy, the patient developed profuse, watery diarrhea. He was diagnosed with *Clostridioides difficile* colitis, which was treated with oral vancomycin. However, despite receiving adequate treatment for the colitis, he developed a toxic megacolon requiring exploratory laparotomy with ileostomy. Daptomycin 500 mg every 48 h was added to have extensive Gram-positive coverage.

Forty-eight days after admission, he was discharged to a skilled nursing facility to complete an additional 48-day empiric antibiotic therapy, as no pathogen was identified by microbiological analysis. He was followed at the outpatient clinics by serial spine imaging until abscess resolution was shown. By the 12-month follow-up evaluation by our team, his strength improved after physical therapy.

## Discussion

Intramedullary spinal cord abscess is a rare diagnosis of the central nervous system. Approximately 200 cases have been reported in the literature [1,2,4]. ISCA may occur in immunocompromised patients due to the secondary spread of an ongoing infection [5]. Congenital spine abnormalities, such as congenital dermal sinus and other spinal deformities, are associated with an increased risk of developing ISCA in children [6]. The presentation may be acute, subacute, or chronic. Patients with ISCA are classified as acute if they have symptoms for less than a week, subacute if they have symptoms for one to six weeks, and chronic if symptoms have been present for more than six weeks. Patients who present acutely are more likely to have a bad prognosis [3]. Rapid surgical intervention is recommended due to detrimental progression. As the symptoms mimic the more common tumoral lesions of the spinal cord, intramedullary abscesses frequently go unnoticed [6].

Early diagnosis, rapid decompressive laminectomy, abscess drainage, and appropriate antibiotic therapy are of extreme importance to improve prognosis. Al Barbarawi et al. noted *Staphylococcus aureus* as the culprit in all their cases, which has been the most common isolated organism, followed by Streptococcus [3]. However, ISCA has also been associated with *Escherichia coli*, Histoplasma, Brucella, Proteus, Bacteroides, Pseudomonas, Listeria, Actinomyces, and Mycobacterium [6]. In adults, the most common source of infection is hematogenous spread; however, a congenital dermal sinus is the primary source in children [3]. Currently, there is no consensus on antibiotics, but some authors think four to six weeks of intravenous antibiotics, followed by two to three months of oral antibiotics, may be adequate [7].

ISCA associated with discitis/osteomyelitis is extremely rare. Derkinderen et al. reported a similar case to ours in which the abscess originated from cervical discitis/osteomyelitis [8]. They documented that their case was the third intramedullary abscess associated with osteomyelitis and epidural abscess. Both prior cases presented by Byrne et al. involved the lumbar spine [9]. Later, other authors have reported four additional cases associated with discitis/osteomyelitis [10-13]. To our knowledge, the current case is the eighth patient with an ISCA associated with discitis/osteomyelitis. Our patient was diagnosed with an ISCA after receiving antibiotics for multiple lumbar paraspinal abscesses. Due to low suspicion and late diagnosis, he suffered progressive deterioration, resulting in impaired activities of daily living and immobility up to becoming bedridden. He suffered a prolonged 48-day hospital stay and developed multiple complications. However, at the skilled nursing facility, his strength significantly improved.

A high index of suspicion and clinical awareness in high-risk patients is essential for early ISCA diagnosis and management [3,7]. In addition to the systemic implications due to the infectious nature of the ISCA, patients are at risk of infarction of neural structures due to a rapid expansion of the spinal cord within the enclosed spinal canal, resulting in permanent, irreversible neurological deficits [3].

## Conclusions

An ISCA may present without fever, chills, or other symptoms of infection. As the symptoms mimic the more common tumoral lesions of the spinal cord, an ISCA frequently goes unnoticed. Surgical abscess drainage is necessary in most cases to improve the symptoms. If untreated, the patient can present detrimental progression. Early diagnosis, prompt abscess drainage, and appropriate antibiotic therapy are essential to improve prognosis and minimize permanent neurological deficits. Although rare, vertebral osteomyelitis and discitis may progress and develop an ISCA.
